# An overview of thoracic actinomycosis: CT features

**DOI:** 10.1007/s13244-012-0205-9

**Published:** 2012-12-15

**Authors:** Ji-Yeon Han, Ki-Nam Lee, Jae Kyo Lee, Yun Hyeon Kim, Seok Jin Choi, Yeon Ju Jeong, Mee-Sook Roh, Pil Jo Choi

**Affiliations:** 1 Department of Radiology, Dong-A University Medical Center, 3-1, Dongdaesin-Dong, Seo-Gu, Busan, 602-715 Republic of Korea; 2 Department of Radiology, Yeungnam University College of Medicine, Daegu, Republic of Korea; 3 Department of Radiology, Chonnam National University Hospital, Gwangju, Republic of Korea; 4 Department of Radiology, Busan Paik Hospital, Inje University College of Medicine, Busan, Republic of Korea; 5 Department of Radiology, Busan National University College of Medicine, Busan, Korea; 6 Department of Pathology, Dong-A University Medical Center, Busan, Republic of Korea; 7 Department of Thoracic and Cardiovascular Surgery, Dong-A University Medical Center, Busan, Republic of Korea

**Keywords:** Pulmonary infection, Thoracic actinomycosis, Computed tomography

## Abstract

**Background:**

Thoracic actinomycosis is an uncommon, chronic suppurative bacterial infection caused by actinomyces species, especially *Actinomyces israelii*.

**Methods:**

It is usually seen in immunocompetent patients with respiratory disorders, poor oral hygiene, alcoholism and chronic debilitating diseases.

**Results:**

We illustrate the radiological manifestations of thoracic actinomycoses in various involved areas in the thorax.

**Conclusion:**

Thoracic actinomycosis can be radiologically divided into the parenchymal type, the airway type including bronchiectasis, the endobronchial form, and the mediastinum or chest wall involvement type.

***Teaching Points*:**

- *Important risk factors for thoracic actinomycosis are underlying respiratory disorders such as emphysema and chronic bronchitis.*
- *Different CT patterns can be distinguished in thoracic actinomycosis: parenchymal, bronchiectatic, endobronchial and extrapulmonary.*
- *Typical CT findings in the parenchymal pattern are a central low density within the parenchymal consolidation and adjacent pleural thickening.*

## Introduction

Actinomycetes are branching gram-positive anaerobic bacteria belonging to the Actinomyceataceae family and are commensal in the human oropharynx, gastrointestinal tract and female genitalia. It is a rare infection that in the past has been reported to occur in 1 in 300,000 people per year [1]. The incidence of all forms of actinomycosis has declined markedly in the last 3 to 4 decades [2]. The infection can involve every organ of the body, and pulmonary actinomycosis is the third most common type after cervicofacial and abdomino-pelvic forms. The pulmonary form of actinomycosis constitutes 15 % of the total burden of disease, although estimates of up to 50 % have been reported [3]. Pulmonary actinomycosis has worldwide distribution and has no age or race predilection, although men acquire the disease slightly more often than women (in an approximately 3:1 ratio), and children are rarely affected [4, 5]. A higher incidence of pulmonary actinomycosis has been reported in patients with underlying respiratory disorders such as emphysema, chronic bronchitis, bronchiectasis and any condition in which the lung parenchyma are destroyed by a previous infection. Alcoholism, poor oral hygiene, dental disease, and facial or dental trauma are important risk factors for the thoracic form. Various chronic debilitating diseases and conditions may facilitate respiratory tract infection, including being at risk for aspiration and having diabetes mellitus, neurologic and psychiatric diseases, malnutrition, drug abuse and hiatal hernia [1, 3]. Unlike in many other unusual granulomatous infectious diseases, the infection rate does not seem to be increased in immunocompromised patients, such as those with acquired immune deficiency syndrome, undergoing chemotherapy, or on steroid or immunosuppressive therapy [5, 6].

Detection of ‘sulphur’ granules by gram or histologic staining is the principal method of direct detection of Actinomyces. Histopathology reveals an acute inflammation surrounded by fibrosing granulation tissue. This material contains sulphur granules, colonies of organisms forming an amorphous centre surrounded by a rosette of clubbed filaments. Direct bacterial confirmation by culture is difficult because of inadequate anaerobic culture, prior antibiotic therapy or overgrowth of concomitant organisms. Thus, culture of expectorated sputum or bronchoscopy aspirates are usually not successful, while fine-needle aspiration, transbronchial biopsy and computed tomography or ultrasound-guided biopsies lead to accurate diagnoses [1, 4, 7].

The clinical manifestation of the disease has changed to a less aggressive form compared to the pre-antibiotic era. The usual presentation is now an indolent, slowly progressive pneumonia with fever, weight loss, cough, sputum and chest pain. The symptoms and clinical and radiologic signs mimic malignancy or tuberculosis [1]. If the disease progresses to the proximal airway and vessels, life-threatening complications such as massive haemoptysis or bronchoesophageal fistula may occur. The course of the disease depends largely on adequate and early antibiotic therapy. The principal treatment of actinomycosis is long-term use of high-dose intravenous penicillin. However, many recent studies have reported that the short-term treatment is successful and that the traditional intensive, long-term regimen is not necessary [8, 9]. In the setting of abscess, percutaneous drainage in combination with medical therapy may be an option [2]. Surgical resection may be a valid option for patients who do not respond to antibiotics for up to 12 weeks according to a recent study [8].

An awareness of the typical imaging findings of thoracic actinomycosis helps in making an early diagnosis, preventing fatal complications and unwarranted surgery. This pictorial essay reviews the radiological type of thoracic actinomycosis.

## Parenchymal actinomycosis

**Fig. 1:**
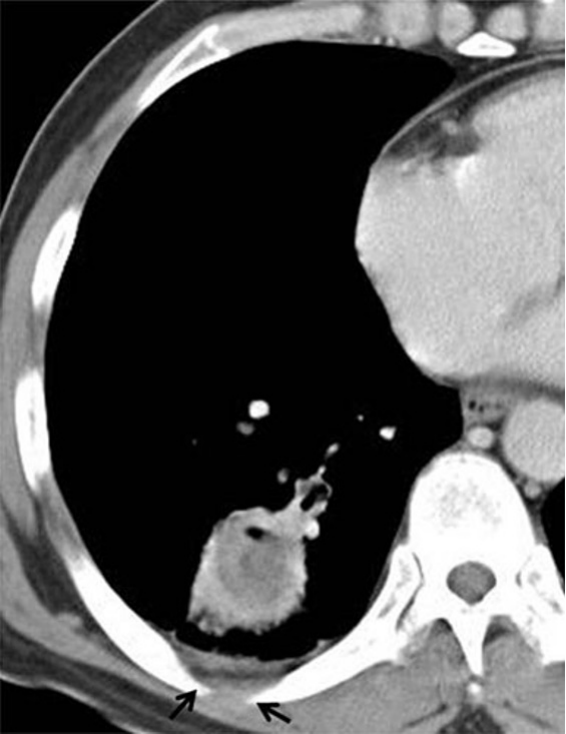
Typical parenchymal actinomycosis in a 58-year-old man with haemoptysis for a month. The CT scan in the mediastinal window setting shows a 3-cm mass-like consolidation with central, low-density peripheral rim enhancement in the right lower lobe and adjacent pleural thickening with subpleural fat infiltration (*arrow*)

**Fig. 2:**
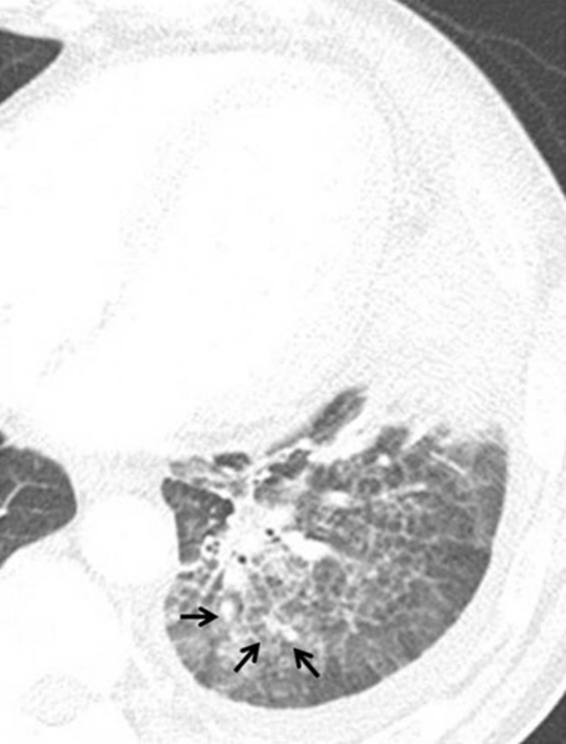
Early stage parenchymal actinomycosis in a 44-year-old man who was a heavy alcohol drinker. The CT image displayed on lung window settings shows focal consolidation surrounded by ill-defined, peripheral pulmonary nodules (*arrows*), which suggests bronchogenic spread of the disease and interlobular septal thickening

**Fig. 3:**
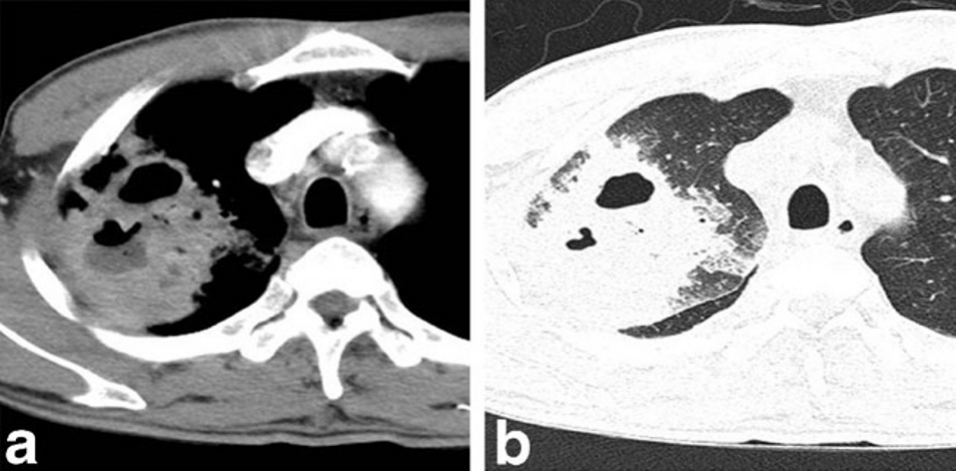
Parenchymal actinomycosis with abscess formation in a 51-year-old man with cough and dyspnoea who had a history of alcoholism. Chest CT scan displayed on a mediastinal setting (**a**) and thin-section CT (**b**) show peripheral consolidation with multiple internal cavities, a central low-attenuation area and adjacent pleural thickening in the right upper lobe

**Fig. 4:**
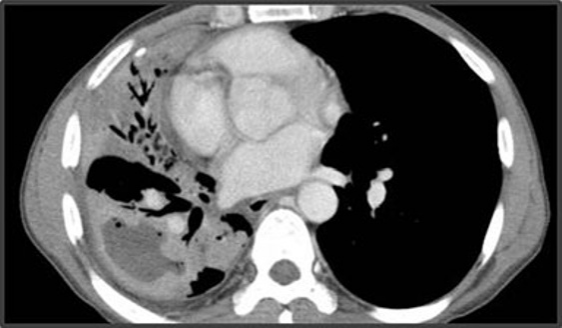
Transfissural extension of parenchymal actinomycosis in a 43-year-old man with high fever for 10 days manifesting as chronic necrotising pneumonia. Chest CT scan in mediastinal window setting shows multifocal lobar consolidation containing a large low attenuation area in the right lower and middle lobe, which demonstrates extension across the fissure

Parenchymal patterns of pulmonary actinomycosis include a peripheral pulmonary nodule, mass or consolidation, all of which may or may not be cavitary or multifocal. Typical CT findings are reported as central areas of low attenuation within the consolidation in 62–75 % of cases and adjacent pleural thickening in 50–73 % (Fig. 1) [10, 11]. The disease initially manifests as a small, poorly defined, peripheral pulmonary nodule with or without interlobular septal thickening. The pulmonary nodule gradually increases to segmental air-space consolidation, which suggests bronchogenic spread of the disease (Fig. 2) [12]. If therapy is not adequate, central areas of low attenuation with a cavity form with the slow progression (Fig. 3). Central low-attenuation areas may be multiple and variable in size and show rim-like peripheral enhancement on enhanced CT [11]. In later stages, lung parenchyma may be destroyed and the infection may extend across fissures to a neighbouring pulmonary lobe (trans-fissural extension), the pleura or chest wall, with abscess formation in these areas (Fig. 4) [10, 11, 13].

Not uncommonly, the infection presents as a mass, sometimes cavitating, that may mimic lung cancer [10, 14]. Associated findings are interlobular septal thickening, bronchiectasis, mediastinal lymphadenopathy, adjacent pleural thickening, pleural effusion and empyema [10, 12, 14].

Histologically, central low-attenuation areas seen on CT represent microabscesses, which contain actinomycotic or sulphur granules. The peripheral enhancing portion is composed of an outer rim of granulation tissue and fibrosis.

The differential diagnosis of the parenchymal type should include tuberculosis, bacterial or fungal necrotising pneumonia, and lung cancer.

## Bronchiectatic actinomycosis

**Fig. 5:**
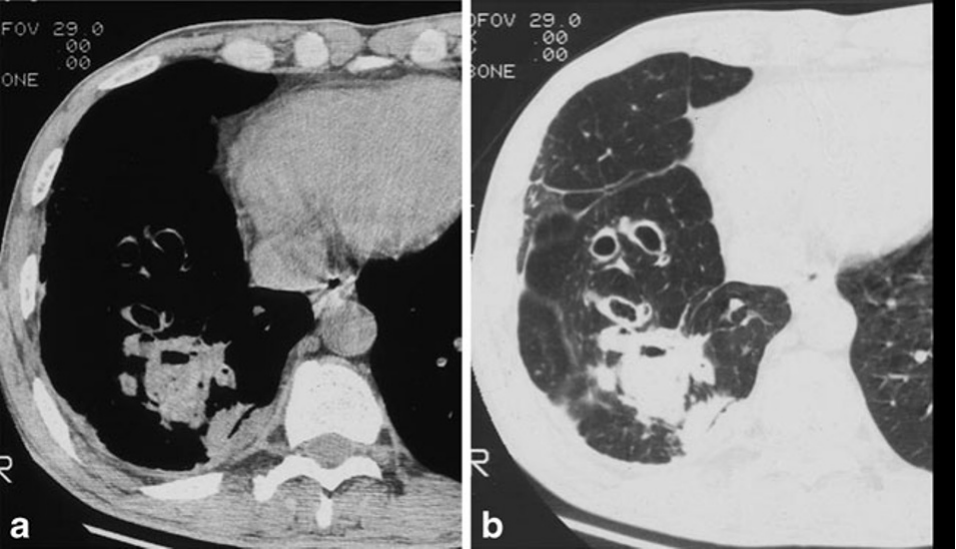
Bronchiectatic form of actinomycosis in a 44-year-old man with underlying bronchiectasis. Chest CT scan displayed on the mediastinal (**a**) and lung (**b**) window setting shows bronchial dilatation with wall thickening and focal consolidation. Note the adjacent pleural thickening in the right lower lobe

**Fig. 6:**
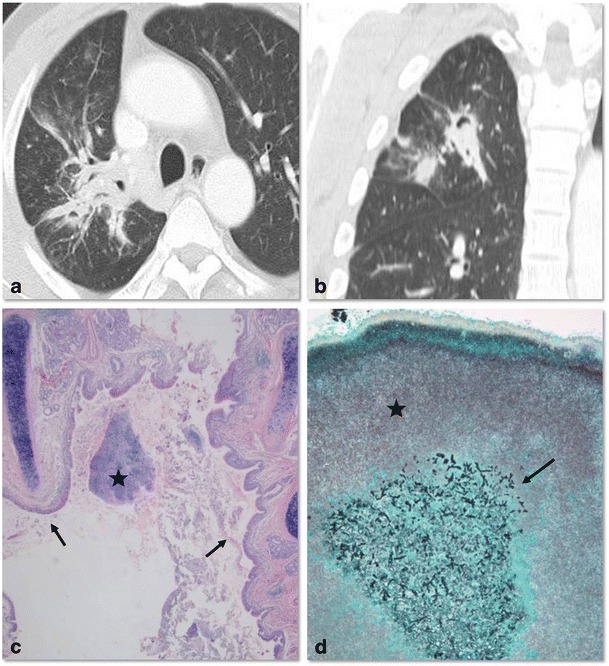
Co-infection of bronchiectatic actinomycosis with Aspergillus in a 52-year-old man. Chest CT scan in lung window setting obtained with an axial scan (**a**) and a coronal reformatted image (**b**) shows focal bronchiectatic changes with intraluminal nodules in the right upper lobe. The presumptive diagnosis was aspergilloma within bronchiectasis. Low-power photomicrography of the surgical specimen from the right upper lobe (**c**) (original magnification, ×20; haematoxylin-eosin stain) demonstrates an Actinomyces colony (*star*) within the ectatic bronchus (*arrows*). Medium-power photomicrograph (**d**) (original magnification, ×400; Gomori methenamine-silver stain) shows a nodule within bronchiectasis composed of central aggregation of Aspergillus (*arrow*) and surrounding Actinomyces colonies (*star*)

**Fig. 7:**
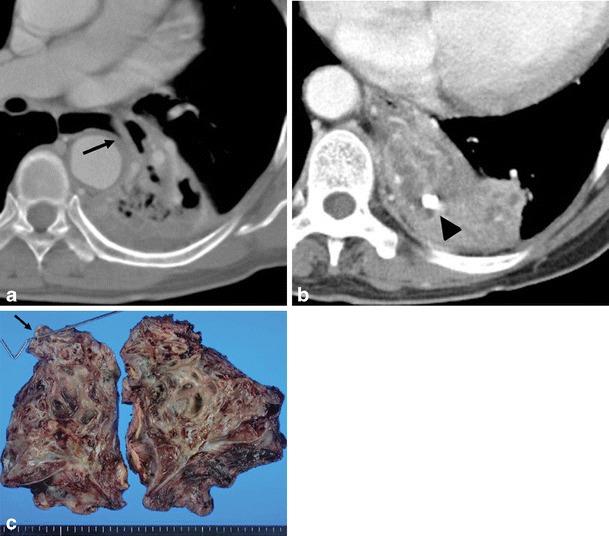
Mixed parenchymal, bronchiectatic and mediastinal actinomycosis in a 69-year-old woman with haemoptysis. **a** Chest CT scan with a modified window setting shows lobar consolidation with cavitary change in the left lower lobe. Note the distended oesophagus with a beak-like appearance that extends toward the consolidation of the left lower lobe (*arrow*). This lesion was confirmed to be an oesophagobronchial fistula after lobectomy. **b** Chest CT scan with the mediastinal window setting at the level below (**a**) shows calcified brocholith (*arrowhead*) within the dilated tubular mucus retained bronchus. **c** Left lower lobectomy specimen showed cystic dilation of thickened bronchi, filled with greyish, fragile and granular material with a foul odour. A connection between the oesophagus and bronchus (oesophagobronchial fistula, *arrow*) was found where the steel rod is pointing (*arrow*)

Actinomyces has a tendency to colonise devitalised tissue [15]. Actinomyces spp. colonise in the dilated bronchi and exacerbate pre-existing bronchial inflammation and bronchiectasis. Previous infections resulting in lung destruction such as tuberculosis and bacterial infections predispose patients to actinomycosis (Fig. 5). Common co-pathogens for thoracic actinomycosis have been described as Actinobacillus actinomycetemcomitans, Staphylococci, Streptococci, Haemophilus spp. and Aspergillus [16, 17]. The pathogenesis of co-infection is a synergistic effect: oxygen deprivation due to other bacteria creates an anaerobic milieu in which actinomyces thrive (Fig. 6) [16]. CT features of the bronchiectatic form include localised areas of bronchiectasis, irregular bronchial wall thickening, and irregular peribronchial consolidation with or without abscess formation [12]. The parenchymal type and bronchiectatic form may occur simultaneously. It is sometimes difficult to differentiate cystic bronchiectasis from central low attenuated necrotic areas of the parenchymal type (Fig. 7).

## Endobronchial actinomycosis

In rare cases infection can cause an endobronchial infection. The endobronchial form reflects actinomycosis colonisation of pre-existing obstructive broncholiths or endobronchial foreign bodies, which inflames the adjacent airway and causes distal obstructive pneumonia. Broncholiths are formed by erosion of calcified lymph nodes into the airway as a result of a granulomatous process. The most common granulomatous infections associated with broncholithiasis include *Mycobacterium tuberculosis* and *Histoplasma capsulatum* [17]. Other causes of broncholithiasis include aspiration of bone or foreign material, erosion by and extrusion of calcified bronchial cartilage plates [18].

**Fig. 8:**
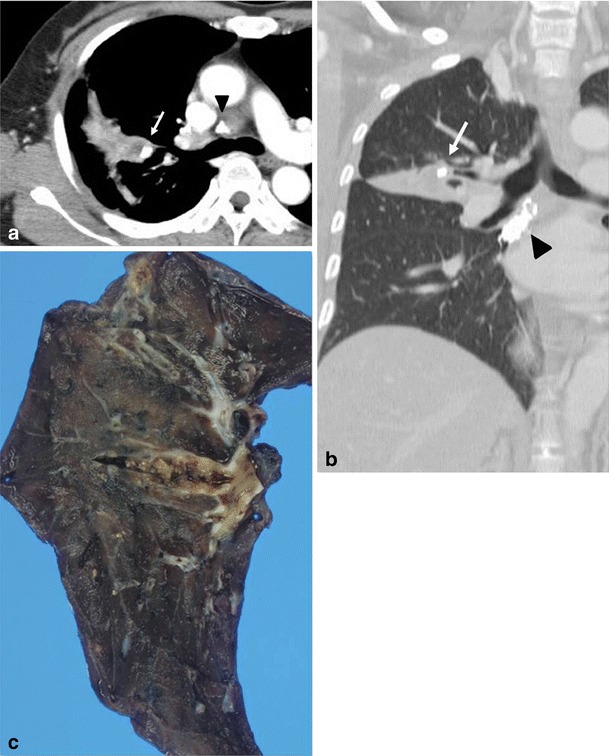
Endobronchial actinomycosis with broncholithiasis in a 53-year-old woman. **a**–**b** The axial and coronal chest CT scans in the lung window setting show a small broncholith (*arrow*) obstructing the lumen of the subsegmental bronchus of the anterior segment and distal subsegmental consolidation within the internal necrotic portion. Broncholiths were confirmed as granular colonies of Actinomyces. Calcified mediastinal lymph nodes are also noted, suggesting previous tuberculosis infection (*arrowheads*). **c** Photographs of the right upper lobectomy specimen show dilatation of central bronchi filled with yellowish-grey granular and fragile material

Actinomyces seem to colonise devitalised tissue and secondarily invade pre-existing broncholiths. Subsequent inflammation enlarges the endobronchial lesion, which leads to obstructive pneumonia. Endobronchial actinomycosis associated with broncholithiasis manifests on CT as a proximal obstructive calcified endobronchial nodule with distal obstructive pneumonia of the involved lobe or segment (Fig. 8) [15]. On the pathological workup, the calcified foci are mixed with actinomyces microcolonies and the distal pneumonic consolidation seen on CT is composed of acute suppurative inflammation, abscesses or organising pneumonia [15].

Bronchial infection may occur from aspiration of a foreign body contaminated with Actinomyces organisms. This type of infection develops in debilitated patients with poor oral hygiene and in a condition that facilitates foreign body aspiration. Reported causes of aspirated foreign bodies resulting in thoracic actinomycosis include teeth, chicken bones, fish bones, grape seeds and beans. None of the cases were associated with choking [19]. CT shows a radio-opaque foreign body with surrounding bronchial thickening and distal obstructive pneumonia. Other secondary findings such as consolidation, atelectasis, bronchiectasis and pleural effusion may also be present [12, 19]. Calcified endobronchial actinomycosis should be differentiated from an aspirated foreign body or de novo calcification. Broncholiths are usually associated with evidence of previous tuberculous infection such as calcified hilar and mediastinal lymph nodes, and the calcification conforms to the shape of the bronchial lumen [20]. These features may be absent with foreign body aspiration.

Rare case reports of endobronchial actinomycosis are associated with an endobronchial mass. In these cases, a reddish polypoid friable mass within the bronchi and surrounding inflamed mucosa is demonstrated by bronchoscopy. CT may not show a distinguishable endobronchial mass but a narrowed bronchus and atelectasis. These endobronchial forms may mimic endobronchial tuberculosis or bronchogenic carcinoma [21, 22].

## Extrapulmonary spread

Actinomycosis may spread from the lung to the pleura, mediastinum and chest wall, with little regard for anatomic barriers. The organisms produce proteolytic enzymes, and peripheral pneumonia tends to involve the pleura, producing empyema, and invade the chest wall with involvement of bones such as the ribs or vertebrae [10, 23]. Chest wall involvement is usually preceded by lung infection, but may occur by means of a direct extension from the neck, oesophagus, abdomen or retroperitoneum [14]. Chest wall involvement is much less common now than previously reported because antibiotic treatment earlier in the disease course is now the rule [23].

**Fig. 9:**
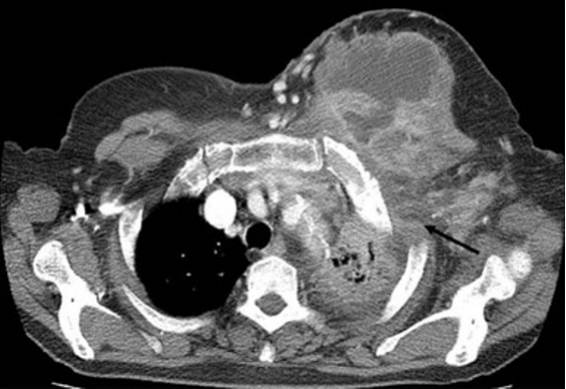
Actinomycosis involving the chest wall in a 75-year-old woman, manifesting as a palpable mass on the left chest wall. Axial CT image shows a heterogeneous mass with central low attenuation and peripheral enhancement on the left chest wall, contiguous with consolidation (*arrow*) in lung parenchyma, and pleural effusion

Radiological manifestations of chest wall involvement include a soft tissue chest wall mass continuous with pulmonary disease, with or without central low-attenuation, empyema, periosteal proliferation along the ribs, and destruction of ribs or vertebrae (Fig. 9) [23]. Further progression of the infection may result in bronchocutaneous fistulas or intercostal fistulas [10]. Pulmonary infections, which can produce similar findings of contiguous chest wall invasion, are tuberculosis, blastomycosis, nocardiosis, cryptococcosis and invasive aspergillosis. Other differential diagnoses are lymphoma, bronchogenic carcinoma, malignant mesothelioma and rare chest wall tumours [23].

**Fig. 10:**
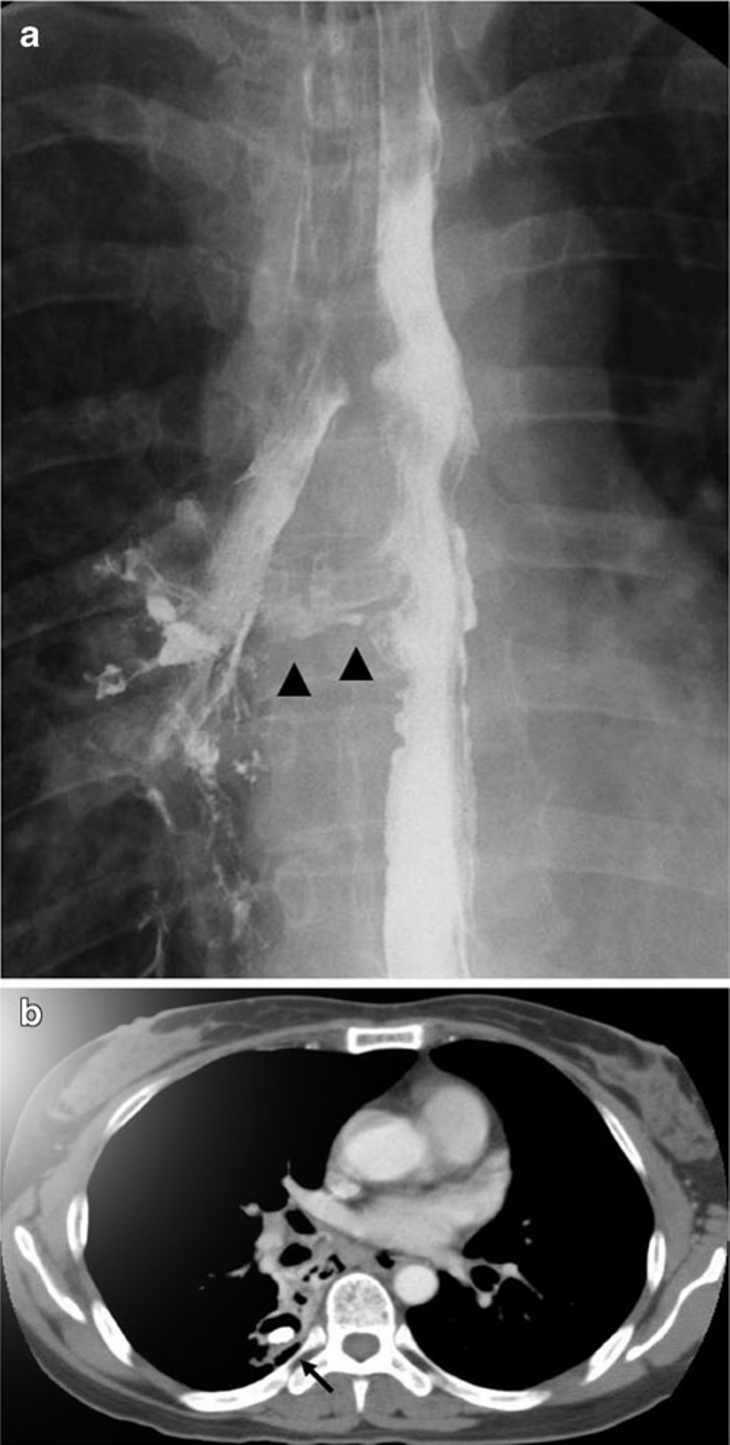
Actinomycosis with oesophagobronchial fistula in a 44-year-old woman who complained of a repetitive cough with food intake. **a** Barium oesophagography depicts the fistula tract (*arrowheads*) between the oesophagus and right bronchus intermedius. **b** Chest CT scan shows a segmental consolidation and bronchiectasis containing a broncholith (*arrow*) in the right lower lobe of the superior segment. Consolidation extends to the mediastinum and oesophagus

Mediastinal actinomycosis is extremely rare. Most cases are cardiac actinomycosis with involvement of the pericardium. Infection most commonly results from contiguous spread from adjacent lung or may be induced by perforation of the oesophagus, chest trauma, thoracic surgery, tracheobronchial perforation or, rarely, hematogenous dissemination [24]. Reported findings are pericardial effusion with or without pleural effusion, a pericardial mass, a mediastinal mass resulting in various complications such as superior vena cava syndrome, Pancoast syndrome or an oesophagotracheal fistula (Figs. 7 and 10) [25–28]. Actinomycotic bacteraemia is very uncommon, but arises more frequently from pulmonary foci than from any other form; it may give rise to metastatic abscesses in other viscera, the skeleton or soft tissues.

## Conclusions

Thoracic actinomycosis may range from subclinical illness to a progressively fatal disease. The prognosis is excellent with low mortality when the disease is recognised early and appropriate treatment is given. Thus, actinomycosis should be considered as part of the differential diagnosis when confronted with chronic unresolving pneumonia that extends across anatomic barriers or manifests as an endobronchial lesion. Early diagnosis may prevent serious complications or unwarranted surgery.
